# Effect of surface treatment and resin cement type on the bond strength of polyetheretherketone to lithium disilicate ceramic

**DOI:** 10.1186/s12903-024-04269-8

**Published:** 2024-05-02

**Authors:** Engy N. Adeeb Gabra, Hoda M. Abdel Sadek, Amina Mohamed Hamdy, Marwa M. Wahsh

**Affiliations:** 1 Lecturer at Fixed Prosthodontics Department, Faculty of Dentistry, Ain Shams University, Cairo, Egypt; 2 Faculty of Dentistry, Ain Shams University, Cairo, Egypt; 3 Fixed Prosthodontics Department, Faculty of Dentistry, Ain Shams University, Galala University, Cairo, Egypt

**Keywords:** PEEK, Surface treatment, Shear bond strength, Veneering material

## Abstract

**Background:**

This study aims to evaluate the effect of surface treatment and resin cement on the shear bond strength (SBS) and mode of failure of polyetheretherketone **(PEEK**) to lithium disilicate ceramic **(LDC**). This is suggested to study alternative veneering of PEEK frameworks with a ceramic material.

**Methods:**

eighty discs were prepared from **PEEK** blank and from lithium disilicate ceramic. Samples were divided into four groups according to surface treatment: Group (**A**) air abraded with 110 μm Al_2_O_3_, Group **(AP**) air abrasion and primer application, Group (**S**) 98% sulfuric acid etching for 60 s, Group (**SP**) Sulfuric acid and primer. Each group was subdivided into two subgroups based on resin cement type used for bonding LDC:1) subgroup (**L**) self- adhesive resin cement and 2) subgroup (**B**) conventional resin cement (*n* = 10). Thermocycling was done for all samples. The bond strength was assessed using the shear bond strength test **(SBS)**. Failure mode analysis was done at 50X magnification with a stereomicroscope. Samples were chosen from each group for scanning electron microscope (SEM). The three-way nested ANOVA followed by Tukey’s post hoc test were used for statistical analysis of results. Comparisons of effects were done utilizing one way ANOVA and (*p* < 0.05).

**Results:**

The highest mean of shear bond strength values was demonstrated in Group of air abrasion with primer application using conventional resin cement **(APB)** (12.21 ± 2.14 MPa). Sulfuric acid groups showed lower shear bond strength values and the majority failed in thermocycling especially when no primer was applied. The failure mode analysis showed that the predominant failure type was adhesive failure between cement and PEEK, while the remaining was mixed failure between cement and PEEK.

**Conclusion:**

The air abrasion followed by primer application and conventional resin cement used for bonding Lithium Disilicate to PEEK achieved the best bond strength. Primer application did not have an effect when self-adhesive resin cement was used in air-abraded groups. Priming step is mandatory whenever sulfuric acid etching surface treatment is utilized for PEEK.

## Introduction

Currently, there is increased interest in metal-free materials such as ceramics, composite resins, and polymers. Ceramics have been widely used due to their inert nature and remarkable esthetics. However, their inherent brittle behavior is still a concern. Consequently, more focus is now directed toward polymeric materials. Polyether-ether ketone (PEEK) has attracted increasing interest because it has desirable properties for dental applications [1].

PEEK is a member of the poly aryl ether ketone (PAEK) family of high-performance polymers that comprises aromatic benzene molecules linked by ether and ketone groups. PEEK is amenable to changes that allow for an increase in the number of characteristics suitable for its application. Sulfonation, nitration, and amination are examples of chemical alterations. Fillers such as ceramic particles, glass beads, or carbon fibers can also be added [2]. PEEK enhanced with 0.3–0.5 µm ceramic fillers improves the material’s mechanical, biological, and physical characteristics, making it particularly effective for dental applications. It has been utilized in implants, temporary abutments for implant-supported prostheses, healing abutments, implant -supported bars, clamp material, and frames of removable and fixed partial dentures. When replacing distal extension cases, it is thought to be an excellent alternative material for abutments with low periodontal support. The material has a low elastic modulus, allowing it to function as a stress breaker and decrease the forces imparted to the repairs [3, 4].

PEEK veneering is essential for improving the aesthetic results of restorations. The grayish white and opaque appearance of PEEK precludes its application as a monolithic repair material. To achieve acceptable aesthetic outcomes, PEEK substructures have typically been veneered with methyl methacrylate (MMA)- or di-methacrylate (DMA)-based materials and ceramic-based restoratives [5, 6].

The inert nature of PEEK is a particularly important trait in terms of biological behavior; nonetheless, it creates a significant barrier in regard to bonding to the material. To obtain the bonding potential of the PEEK surface, several surface modifications tend to be required [7]. Surface treatment is the practice of adjusting the surface features of materials to improve their biological and mechanical capabilities without changing their overall properties. Increasing the bond strength between resin cement and prosthetic material necessitates both micromechanical locking and chemical bonding [8, 9]. There are two methods to achieve an enhanced bonding performance of PEEK: the alteration of surface topography and conditioning with an adhesive system to enable chemical interactions. Multiple studies have examined the bond strength between resin and PEEK materials using different pretreatment methods, such as air abrasion, silica coating [9–11], sulfuric acid etching [10, 12], piranha etching [13], laser treatment [14], different types of plasma [15] or photodynamic therapy (PDT) [16]. Air abrasion and sulfuric acid etching surface treatment are among the most commonly used surface treatment methods for enhancing PEEK bonding to resin-based materials [17–19]. The application of chemical conditioners for inert polymeric materials before veneering and cementation protocols is desirable [4, 11, 17, 20]. Previous studies have linked the use of bonding agents with DMA, MMA, or pentaerythritol triacrylate (PETIA) in their chemical makeup to higher bond strength [11, 21]. However, there is still conflict of data regarding the suitable surface treatment of PEEK polymers for achieving better bonding with different classes of resin cement.

Lithium disilicate ceramic materials have proven successful and outstanding results in their dental application as prosthetic materials. It offers superior mechanical and optical properties. After surface treatment via hydrofluoric acid etching and silane application, the material has been investigated in detail, and it has been shown to have optimal bonding potential with resin-based materials [6, 22]. Therefore, this material is suggested as an alternative to the conventional veneering of PEEK. Ceramic materials have proven successful in overcoming the drawbacks of composite resin materials in terms of durability, color stability and wear resistance [23, 24]. There is limited research on the veneering of PEEK with ceramic materials. As a result, the purpose of this study was to investigate the feasibility of employing a ceramic material to veneer the PEEK polymer as an alternative to standard composite veneering after different surface treatments of PEEK and using diverse types of resin cement. The null hypothesis states that the various surface treatments and resin cements will not have a significant influence on the shear bond strength between PEEK and the ceramic material.

## Materials and methods

The materials used in this study, lot numbers, manufacturing methods and compositions are listed in Table 1.

**Table 1:** The materials, lot number, manufacturer, and composition

Material	Lot number	Manufacturer		Composition
breCAM. BioHPP (PEEK)	482,047	Bredent GmbH&Co Germany		Polyetheretherketone 80%, Fillers: Aluminum oxide & zirconium Oxide (0.3–0.5 μm) 20%, Pigments (Ti, Ni, Sb) O_2_ ≤ 1%.
Visio. Link (Light-curing primer)	214,511	Bredent GmbH&Co Germany		MMA ^(^Methyl methacrylate), PETIA (pentaerythritol—Tri acrylate), photo initiator
IPS Emax Press. Lithium disilicate glass- ceramic	Z02GJ8	Ivoclar Vivadent, Schaan, Liechtenstein		SiO_2_ 57.0–80.0%, Li_2_O 11.0–19.0%, K_2_O 0.0–13.0%, P_2_O_5_ 0.0–11.0%, ZrO_2_ 0.0–8.0%, ZnO 0.0–8.0%, other and coloring oxides 0.0–12.0%
Sulfuric acid	123022S98	Alfa chemical Group ACG-Egypt		98% sulfuric acid
Bisco Porcelain Etchant	2,000,007,009	Bisco Schaumburg, USA.	Inc. IL,	9.5% buffered Hydrofluoric acid gel
Bisco porcelain Primer Silane coupling agent	2,000,006,994	Bisco Schaumburg, USA.	Inc. IL,	Pre hydrolyzed silane with methacrylate 1– 10%’ Ethanol, 30–70; acetone, 30–70
Duo-Link Universal Adhesive resin Cement	2,300,001,015	Bisco Schaumburg, USA.	Inc. IL,	Bis-GMA (Bisphenol A diglycidylmethacrylate), TEGDMA (Triethylenglycol Dimethacrylat, Glass filler.
All-bond Universal	2,200,000,398	Bisco Schaumburg, USA.	Inc. IL,	10-MDP (methacryloyloxydecyl dihydrogenphosphate), 2-HEMA (Hydroxyethyl Methacrylate), BisGMA, ethanol, water, photoinitiator
Bis Cem Self- adhesive resin cement	2,200,000,549	Bisco Schaumburg, USA	Inc. IL,	BisGMA, UDMA, TEGDMA, HEMA, 4META resins, silane-treated barium borosilicate glasses, silica with initiators, stabilizers, and UV absorber, organic and/or inorganic pigments, opacifiers

A power analysis was designed to have adequate power to apply a statistical test of the null hypothesis that no difference would be found between different tested groups regarding shear bond strength. By adopting an alpha level of (0.05), a beta of (0.2) (i.e., power = 80%) and an effect size (f) of (0.503) calculated based on the results of a previous study [14], the predicted sample size (n) was 10 samples per subgroup. The sample size was calculated using G*Power version 3.1.9.7 [25].

### 1-Sample grouping

 The samples were first divided according to the type of surface treatment and conditioning with the PEEK primer into four groups: group (**A**) air abraded with 110 μm Al_2_O_3_; group **(AP**) air abrasion and primer application; group (**S**) 98% sulfuric acid etching for 60 s; and group (**SP**), which included sulfuric acid and primer. Each group was subdivided into two subgroups based on the resin cement type used for bonding LDC: (1) subgroup (**L**) was self-adhesive resin cement, and (2) subgroup (**B**) was conventional resin cement (*n* = 10).

### 2- PEEK sample fabrication and surface treatments

**Fig. 1:**
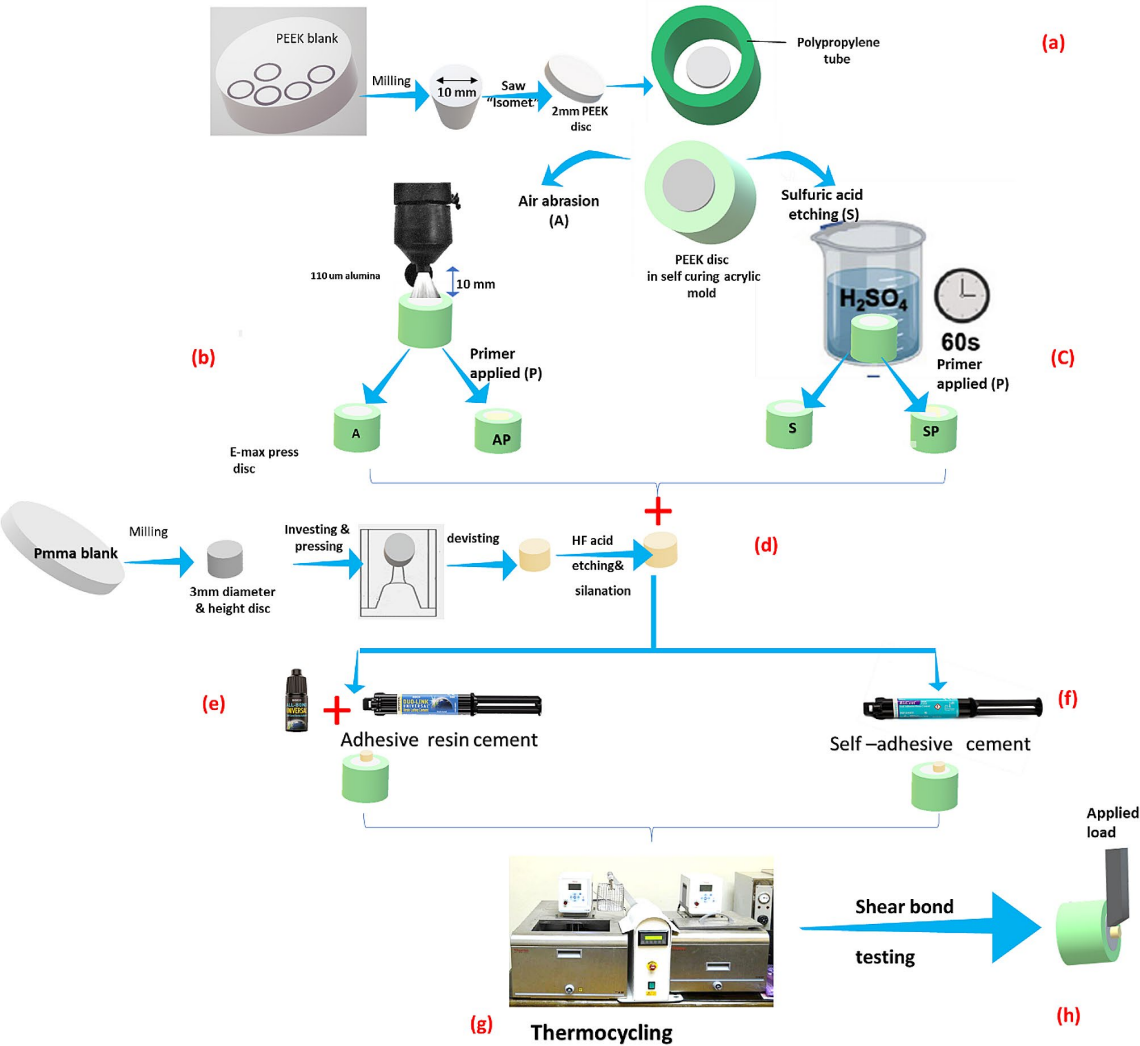
Flowchart for the specimen preparation, aging, and testing protocol; a: PEEK sample fabrication, b: air abrasion surface treatment, c: sulfuric acid surface treatment, d: IPS e-max sample fabrication, e: bonding with conventional resin cement, f: bonding with self-adhesive resin cement, g: thermocycling aging, h: shear bond strength

Cylinders with a diameter of 10 mm were designed with 3D builder software (Microsoft Corp, USA) and milled out of the bre-Cam BioHPP blank (Bredent GmbH & Co KG) by means of a VHFS5 5-axis milling machine (Ammerbuch, Germany) (Fig. 1a). Each cylinder was then cut into 2 mm thick discs of PEEK by means of a low-speed diamond saw under a water coolant (IsoMet™ 5000 Buehler, USA). 80 PEEK discs (10 mm diameter and 2 mm thickness) of breCAM. BioHPP (Bredent GmbH & Co KG) were sectioned. The samples were embedded in an autopolymerizing acrylic resin (Acronstone, Egypt) mold. The bonding surfaces were polished with 600- and 800-grit silicon carbide paper under running water in circular motion for 30 s for standardization purposes. Polished samples were then ultrasonically cleaned (Durasonix 3.2 L Ultrasonic Cleaner, China) in a distilled water bath for 10 min. The samples were randomly divided into 4 groups (*n* = 20) according to the PEEK surface treatment (Fig. 1b and c). The detailed surface treatments for the distinct groups are listed in Table 2.

**Table 2:** Surface treatments for PEEK discs

Surface treatment	Procedures
Air Abrasion	The sandblasting procedure was applied with 110 Al_2_O_3_ particles (Cobra Aluminum. Renfert) at 2.5 bar pressure,10 mm distance perpendicular to polymer specimens, for 10 s
Air Abrasion followed by primer application	Samples were air abraded as mentioned then Visio. Link PEEK primer was applied by means of a micro brush. A single application was done followed by light curing in bre. lux power unit 2 (Bredent, Germany) for 90 s. Obtaining a semimatt surface finish indicated optimal primer thickness as indicated by manufacturer.
Sulfuric acid	The surface of the PEEK sample was etched by immersion in 98% sulfuric acid solution (Sprea Misr, Egypt) for 60 s, rinsed with distilled water for 60 s, and air-dried with oil- free air.
Sulfuric acid followed by primer application	Samples were etched as mentioned then Visio. Link PEEK primer was applied in the same way of air abraded group.

### 3- Ceramic sample fabrication

Lithium disilicate ceramic (LDC) discs 3 mm in diameter and 3 mm in height were obtained using pressing technology with IPS e-max press ingots (Ivoclar Vivadent, Schaan, Liechtenstein) (Fig. 1d). Dimensions were confirmed using a digital caliber (Mitutoyo Digimatic caliper, USA). LDC samples were surface treated according to the manufacturer’s recommendation. The samples were etched with 9.5% hydrofluoric acid (Bisco, USA) for 20 s, rinsed, and air-dried. Ceramic primer (silane) (Bisco, USA) was applied by a brush for 10–12 s, and the samples were gently air-dried.

### 4- Bonding procedure

Two types of resin cement were used to bond LDC to the PEEK surface: self-adhesive and conventional resin (Fig. 1e and f). For bonding using conventional resin cement, a bonding agent (Bisco, USA) was applied separately, and the sample was cured with LED curing unit (Woodpecker Led-F, China) at a minimal intensity of 1200 mw/cm^2^ for 20 s prior to applying the conventional resin cement. Resin cement was dispensed on the PEEK disc, and then the ceramic disc was fixed in place with finger pressure. Ramp curing of the resin cement was performed for 5 s, and the excess material was removed. Light curing was completed by means of LED curing for 20 s circumferentially using LED curing unit (Woodpecker Led-F, China) at a minimal intensity of 1200 mW/cm^2^. For bonding with self-adhesive resin cement, the same steps were performed without the application of a bonding agent.

### 5- Aging with thermocycling

Then, the samples were stored in distilled water at 37 °C and subjected to a thermocycling aging process for 5000 thermocycles in a thermal cycling simulation machine (Thermocycler-THE1100, SD Mechatronics, Feldkirchen-Westerham, Germany) between 5 °C and 55 °C in water with a 30 s dwell time (immersion time in each bath) and a 10 s transfer time between baths (Fig. 1g).

### 6- Shear bond strength test (SBS test)

SBS tests were performed using a universal testing machine (Model 2719 − 113; Instron Corp., Norwood, MA). A chisel-shaped blade was directed at the bonding interface at a 1 mm/min crosshead speed. The tested samples were positioned parallel to the loading direction of the jig of the testing machine (Fig. 1h). The maximum force (Newton) was recorded, and the following formula was used to calculate the SBS data: fracture load/bonding surface area (pi*R(radius)^2^) = N/mm2 = MPa, as described in the ISO 10477 standards [26].

The samples that did not survive the thermocycling process were observed and considered to have a shear bond strength of zero. Failure mode analysis was performed at 50X magnification with a stereomicroscope (Zeiss Discovery V20; Zeiss) and described as adhesive (between the PEEK and resin material), cohesive, or mixed (both adhesive and cohesive failures occurred). Samples were chosen from each group for scanning electron microscopy (SEM) (Thermo Scientific™ Quattro ESEM) observation.

Numerical data are presented as the mean and standard deviation (SD). The data were checked for normality by observing the data distribution and using the Shapiro‒Wilk test. The data were considered to be normally distributed, and three-way nested ANOVA followed by Tukey’s post hoc test were used for analysis. Comparisons of simple effects were performed utilizing one-way ANOVA and the pooled error term of the three-way model. *P* values were adjusted for multiple comparisons utilizing Bonferroni correction. The significance level was set at *p* < 0.05. Statistical analysis was performed with R statistical analysis software version 4.3.0 for Windows (R Core Team (2023)). R: A language and environment for statistical computing. R Foundation for Statistical Computing, Vienna, Austria).

## Results

Failure of bond during the thermocycling process comprised 100% of the SB & SL group and 20% of the SPL group, so shear bond strength values for the SB and SL groups were excluded and considered not applicable (**NA**) for statistical comparisons. Three-way ANOVA showed that surface treatment and resin cement type had significant effects on the shear bond strength. The interactions between the independent variables, surface treatment, resin cement and PEEK primer placement, had a significant effect on the shear bond strength (Table 3). The mean and standard deviation (SD) values of the shear bond strength (MPa) for different surface treatments and for different resin cements within other variables are presented in Table 4**and** Table 5. One-way ANOVA followed by Tukey’s post hoc test showed that air-abraded samples with the PEEK primer had a significantly greater effect than acid-etched samples with the PEEK primer in both types of resin cement. samples (*p* < 0.00) (Table 4). In addition, in the air abrasion group with primer applied, the Adhesive resin cement (12.21 ± 2.14) was significantly greater than that of self-adhesive resin cement (7.92 ± 0.66) (*p* < 0.001) (Table 5).

**Fig. 2:**
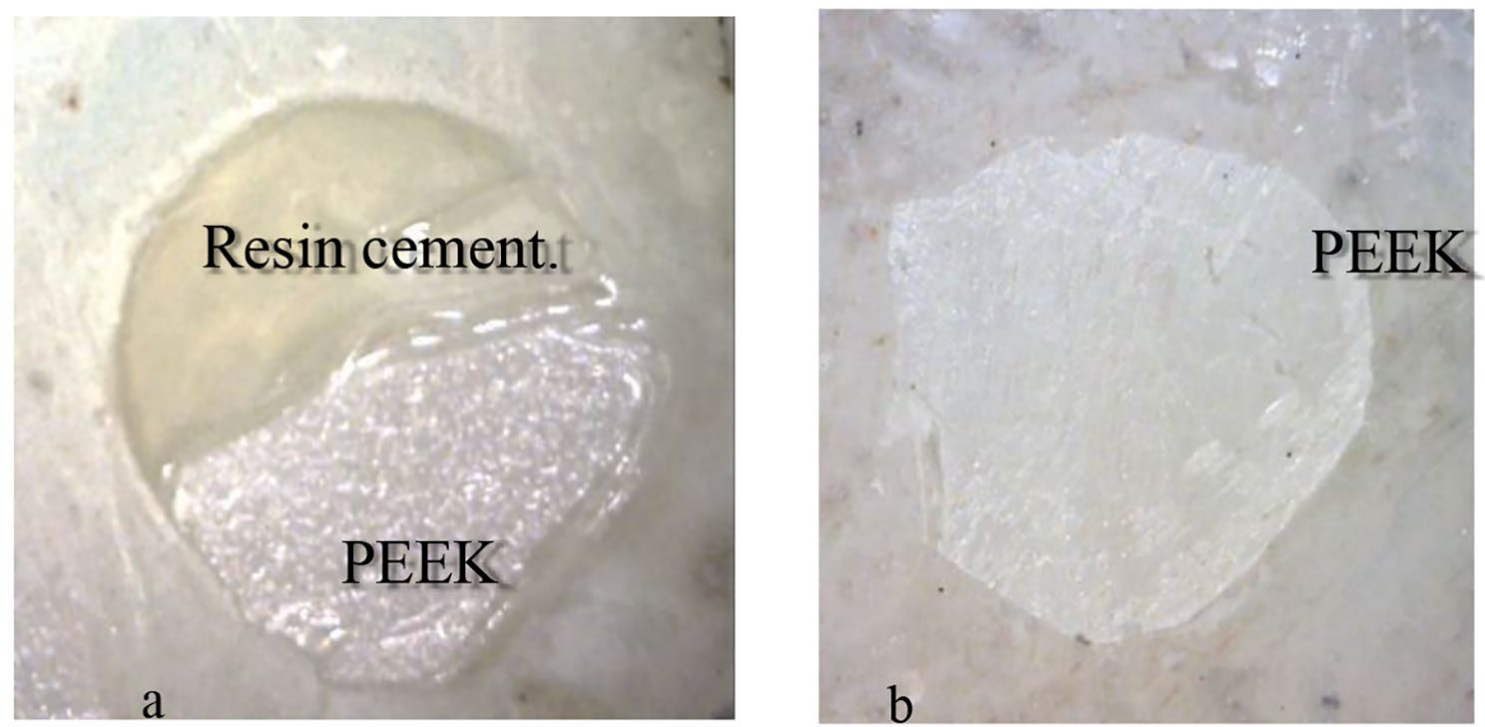
Failure mode; **a**: mixed failure, **b**: adhesive failure

The failure mode analysis of all the experimental groups is shown in Table 6. There was no complete cohesive failure in either the ceramic or resin cement (0%). The predominant failure type was adhesive failure between the cement and PEEK, while the remaining failure type was mixed failure between the cement and PEEK (Fig. 2).

**Table 3:** Effect of different variables and their interactions on the shear bond strength (MPa)

Variable	Sum of Squares	df	Mean Square	f value	*p* value
**Surface treatment**	335.36	1	335.36	73.72	< 0.001*
**Resin cement**	72.64	1	72.64	15.97	< 0.001*
Surface treatment * Resin cement	0.00	1	0.00	0.00	**0.975ns**
Surface treatment * resin cement*PEEK primer	31.00	2	15.50	3.41	0.043*

df = degree of freedom*; significant (*p* ≤ 0.05); ns, nonsignificant (*p* > 0.05)

**Table 4:** Mean and standard deviation (SD) values of the shear bond strength (MPa) for different surface treatments within other variables

Resin cement	PEEK primer	Shear bond strength (MPa) (mean ± SD)		*p* value
		Air abrasion	Acid etching	
**Adhesive**	**With**	12.21 ± 2.14	5.29 ± 3.25	< 0.001*
	**Without**	9.62 ± 2.23	**NA**	**NA**
**Self-adhesive**	**With**	7.92 ± 0.66	2.85 ± 1.92	< 0.001*
	**Without**	8.96 ± 1.75	**NA**	**NA**

NA: Not Applicable, *; significant (*p* ≤ 0.05) ns; nonsignificant (*p* > 0.05)

**Table 5:** Mean and standard deviation (SD) values of shear bond strength (MPa) for different resin cements within other variables

Surface treatment	PEEK primer	Shear bond strength (MPa) (mean ± SD)		*p* value
		Adhesive	Self-adhesive	
**Air abrasion**	**With**	12.21 ± 2.14	7.92 ± 0.66	< 0.001*
	**Without**	9.62 ± 2.23	8.96 ± 1.75	**0.520ns**
**Acid etching**	**With**	5.29 ± 3.25	2.85 ± 1.92	**0.089ns**
	**Without**	NA	NA	**NA**

NA: Not Applicable, *; significant (*p* ≤ 0.05) ns; nonsignificant (*p* > 0.05)

**Table 6:** Number from each group for each type of failure mode

Failure mode Groups	Adhesive	Mixed	Cohesive	Total
**APL**	5	5	0	10
**APB**	5	5	0	10
**AL**	9	1	0	10
**AB**	8	2	0	10
**SPL**	10	0	0	10
**SPB**	9	1	0	10
**SL**	10	0	0	10
**SB**	10	0	0	10
**Total**	66	14	0	80

The SEM-representative.

**Fig. 3:**
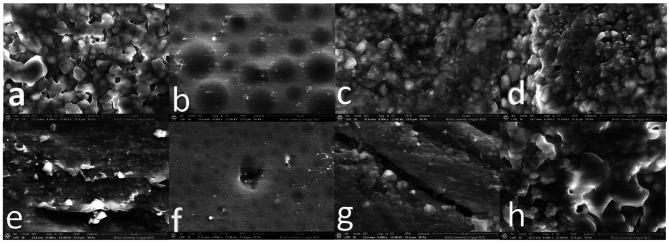
Representative SEM images of PEEK after different surface treatments; **a**: air abrasion, **b**: air abrasion and primer, **c**: air abrasion and bonding agent, **d**: air abrasion and primer and bonding agent, **e**: sulfuric etching, **f**: sulfuric etching and primer, **g**: sulfuric etching and bonding agent, **h**: sulfuric etching and primer and bonding agent

SEM images of the different surface treatments applied are shown in Fig. 3. Air abrasion resulted in surface pitting following alumina air abrasion of the PEEK surface (Fig. 3a). Primer application following air abrasion of the PEEK surface resulted in wetting of the surface, and an evident uniform bubbly pattern was observed (Fig. 3b). The application of the bonding agent to the air-abraded PEEK surface in the **AB** group revealed an absurd surface and irregular wetting of the surface by the resin-based bonding agent (Fig. 3C). The combined application of primer and bonding agent in **APB** resulted in better wetting of the PEEK surface and complete coverage of the surface with an evident resin layer (Fig. 3d). Sulfuric acid etching resulted in evident groove formation on the PEEK surface (Fig. 3e). The application of primers to the acid-etched PEEK surface resulted in wetting of the surface, and an evident bubbly pattern was observed. However, the pattern is different from that of an air-abraded surface, with a smaller bubble form and a less continuous appearance (Fig. 3f). While the application of a bonding agent to the acid-etched PEEK surface in the **SB** group resulted in areas of resin covering the surface. However, poor resin penetration on the surface grooves was evident (Fig. 3g). The combined application of primer and bonding agent in the **SPB** group resulted in an evident surface of the resin covering the surface, but it had multiple cracks and defects, decreasing the uniformity of the surface coverage compared to that of the same applications on the air-abraded group (Fig. 3h).

## Discussion

The null hypothesis was rejected, as statistically significant differences were found among the different tested surface treatments and resin cements used (*P* < 0.05). The SBS values of most test groups (5.29–12.12 MPa) were greater than the lower limit (5 MPa) according to the ISO 10477 standard, and air abrasion combined with primer resulted in a value within the clinically acceptable range (10 MPa), depending on related research [12, 17]. However, the results are still considerably low for clinical application. We reported failure of bonding in sulfuric acid-etched samples when no primer was applied at a percentage of 100% with both conventional and self-adhesive resin cements after thermocycling aging.

Numerous studies have investigated PEEK surface modification methods to enable better bonding to resin-based materials. In this study, two methods for surface modification (air abrasion and sulfuric acid etching) were selected based on the best results obtained from previous research [18, 19].

This in vitro study used ceramic-modified PEEK material in combination with lithium disilicate since the material offers better support for lithium disilicate. This partially crystalline PEEK combines elasticity (approximately 4.200–4.800 MPa), stiffness (flexural strength 180–185 MPa), weight, and breaking strength (from 700 N to 1600 N). The filler strengthening led to improvements in strength and abrasion properties and allowed the material to be veneered [27]. Nevertheless, differences in the flexural strength of the investigated materials should be considered since the flexural strength of lithium disilicate is approximately 340–350 MPa [28]. . Moreover, the cementation of two different elastic materials influences the behavior of the final restoration due to differences in deflection during functional stresses, which can lead to deformation that can negatively affect the bond strength [29].

The bonding between the PEEK surface and lithium disilicate used in this in vitro study depended on the use of two types of resin cement. This was done because there was insufficient data comparing different classes of resin cements combined with different surface treatments of PEEK in cases where lithium disilicate was used [6, 30].

The chosen air abrasion protocol was 110-µm Al_2_O_3_ particles for 15 s at a pressure of 2 bar and a 10 mm distance. It was found that a 110 μm alumina particle size resulted in better surface roughness and enhanced wettability [31, 32]. A concentration of 98% sulfuric acid for the etching of the PEEK surface was selected because this concentration resulted in optimal etching of the PEEK surface with subsequent enhancement of the shear bond values [33].

The use of primer (Visio Link) was targeted in this study to investigate its effect on bonding due to its interaction with the resin cement used. pentaerythritol Tri acrylate, methyl methacrylate, and di methacrylate monomers are all present. It is suggested that pentaerythritol Tri acrylate dissolves the PEEK surface, and subsequently, methyl methacrylate monomers cause swelling of the dissolved surface; eventually, Di methacrylate monomers result in the bonding of the composite resin to the two methyl groups [34, 35].

Thermocycling has proven to be an appropriate method for the simulation of thermal alterations that occur in the oral environment because of eating, drinking, and respiration. It was crucial to subject our samples to such an aging procedure to represent the in vivo conditions that would have a direct influence on the results of the present in vitro study. The selection of thermocycling for five thousand cycles (5 °C/55°C; dwell time, 20 s) corresponds to approximately 4 to 5 years of clinical service. In this way, all tested samples were subjected to standardized and reproducible thermal stress [15, 36, 37].

Compared with the sulfuric acid etching, the air abrasion surface treatment resulted in higher SBS values. This result was in accordance with previous research performed by **Lee et al.** [5]. However, these findings contradict those of previous studies [15] and [32], where sulfuric acid etching yielded better results. SEM revealed significant differences that might explain the results obtained in the present study. The air-abraded PEEK surface evidently had increased surface roughness and more uniform pitting of the surface. This was evident in the study by **Ourahamoun**e [38], who found that mechanical air abrasion creates some sort of beneficial surface roughness for bonding, allowing mechanical locking and penetration of the priming agent along the indentations into the polymer. In addition, the investigations showed that air abrasion readily influenced the hydrophobic behavior of PEEK polymers via modification of the surface morphology and allowed better wetting of the surface. On the other hand, SEM images of the sulfuric acid-etched group showed deep groove formation, which was difficult for the resin to penetrate and hence led to lower SBS values. These results are in accordance with those of **Zhou et al.** [8], who revealed that acid etching caused dense nanoneedle cracks penetrating the basal material. In another study by **Ates et al.** [17], SEM images of the air-abraded group showed an irregular fissure pattern with larger grooves. It was suggested that these surface modifications would increase the contact area for the material to bond with and be more suitable for the flow resin-based materials (i.e., the primer and the resin cement) significantly better, especially when combined with the use of the PEEK primer. This was in line with a study by **Stolarczyk et al.** [11], where the bond strength of PEEK samples bonded to veneering resin was evaluated after various surface treatments and primer application. The highest bond values were found within the air abrasion groups and samples adhesively bonded with Visio. Link chemical primer. Additionally, mixed failure types were found more frequently in the airborne-particle abrasion group when combined with the priming step. It was concluded that the adhesion of the tested PEEK samples was acceptable after treatment with airborne-particle abrasion and when additional adhesive systems such as Visio. Link were used. This was in accordance with previous work by Kern et al. [20], where it was found that only multifunctional methacrylate-containing adhesives on air-abraded PEEK surfaces showed a promising durable bonding to PEEK. In addition, a study by **Hallman et al.** [39]. noted that conditioning the PEEK surface with methyl methacrylate (MMA)-based adhesives following air abrasion enhanced the SBS values. Several studies have noted that the PEEK primer Visio. Link represented the positive control group because of enhanced bonding after the use of Visio. Link as conditioner on various surface-treated PEEK samples reported, and air abrasion was considered one of the best initial surface treatment options for PEEK [40, 41].

The lower shear bond strength values in the sulfuric acid-etched groups came in accordance with a previous study by **Chaijareenont et al.** [33]. It was suggested that the high porosities and rough surfaces on PEEK resulting from 98% sulfuric acid etching may negatively affect the penetration of resin-based adhesives and result in weak points at bond interfaces. Another explanation for the lower bond strength values after sulfuric acid etching might be due to differences in the etched substrate. Our study used modified PEEK material with 20% inorganic filler, while previous studies utilized unfilled PEEK material, which might be more prone to sulfuric acid etching, resulting in greater bonding. In addition, the 98% concentration of sulfuric acid might have been too strong to be used with the type of PEEK material used in this study. Thus, more studies with lower concentrations or shorter etching times might be needed to confirm the validity of this surface treatment modality.

The chemical primer contains MMA and PETIA penetrate the resin matrix of the polymeric material and create entanglements that function as mechanical connections. This is even valid in polymeric restoration materials with a high conversion rate due to industrial curing; many unreacted double bonds are still prevalent. MMA and PETIA allow for covalent bonding to methacrylate in polymers. PETIA specifically leads to an increased crosslinking density at the interface and within the layer of the primer. This can be expected from the high crosslinking density of Visio. link contributes to the good mechanical properties of the interface after curing as well. Another aspect to explain the superior performance of Visio-link might be the good wetting behavior of other polymers, which is a prerequisite for chemical interactions at the interface and for good mechanical interlocking in the micropores of the surfaces [15].

In our study, PEEK primers were most effective when adhesive or conventional resin cement. This may be attributed to the fact that the MDP-containing bonding agent has a hydrophobic methacrylate terminal end copolymerizing with MMA monomers present in the PEEK primer. However, the actual bond strength values were lower in the current study compared to previous studies. This may be attributed to differences in the bonding substrate. In our study, resin cements were used to bond lithium disilicate to the PEEK surface, while previous studies used a veneering resin composite applied directly to the PEEK surface. In addition, it is well established that resin cement is more technique sensitive and more prone to degradation, especially when aging by thermocycling is utilized [42].

Concerning the type of resin cement used, conventional resin cement resulted in higher shear bond strength values in all the groups. However, the results were only significant when the PEEK primer was used, and air abrasion surface treatment was applied. **Sproesser et al.** [43] reported similar data regarding the effect of resin cement type. It was found that the bond strength of conventional resin cement yielded higher bond values compared to the self-adhesive cement. This might be because the use of a highly reactive bonding agent allowed for better wetting of the PEEK-treated surface. In our study, the application of a bonding agent significantly led to higher values when the PEEK primer was used, as a true chemical bond could be easily established between the two resin-based materials and hence had a synergistic effect on the bond values.

The limitations of this study include that only two surface treatments were selected for PEEK bonding to LDC. No comparisons were made between different air abrasion parameters or different concentrations and times for sulfuric acid etching. In addition, no cyclic fatigue has been investigated to study the effect of functional loading of the resulting bond.

## Conclusions

The following conclusions can be drawn according to the obtained results:

1. The combination of air abrasion followed by primer application and conventional resin cement used for bonding lithium disilicate to PEEK achieved the best bond strength. However, the reported value is clinically not as high as desired.
2. Primer application did not have an effect when self-adhesive resin cement was used in the air-abraded groups.
3. The priming step is mandatory whenever a surface treatment involving sulfuric acid etching is utilized for PEEK.

## Recommendations for future studies

Further studies are needed to determine whether sulfuric acid can improve bonding, and it is advisable to apply it to the PEEK surface at a variable concentration and for different periods. Future PEEK surface treatment or cementation methods should aim for higher values of bond strength to improve clinical applications.

## Data Availability

The datasets used and analyzed during the current study are available from the corresponding author on reasonable request.

## Abbreviations

PEEK: polyetheretherketone
LDC: lithium disilicate ceramic
SBS: shear bond strength
A: alumina air abrasion
S: sulfuric acid etching
P: PEEK chemical primer

## References

[1] Wiesli MG, Özcan M (2015). High-performance polymers and their potential application as medical and oral Implant materials: a review. Implant Dent.

[2] Staniland PA, Wilde CJ, Bottino FA, Di Pasquale G, Pollicino A, Recca A (1992). Synthesis, characterization and study of the thermal properties of new polyarylene ethers. Polym (Guildf).

[3] Iyer S, Hegde RSRS, Coutinho DA, Priya C (2020). BioHPP: Properties and Applications in Prosthodontics a review. J Res Dent.

[4] Caglar I, Ates SM, Yesil Duymus Z (2019). An in vitro evaluation of the Effect of various adhesives and surface treatments on Bond Strength of Resin Cement to Polyetheretherketone. J Prosthodont.

[5] Lee KS, Shin MS, Lee JY, Ryu JJ, Shin SW. Shear bond strength of composite resin to high performance polymer PEKK according to surface treatments and bonding materials. (2017) J Adv Prosthodont. 1;9(5):350–7, 10.4047/jap.2017.9.5.350 10.4047/jap.2017.9.5.350 PMC5673611 29142642

[6] Kiliç M, Dede DÖ, Küçükekenci AS. Comparing the shear bond strength of veneering materials to the PAEKs after surface treatments. (2023B) MC Oral Health.;1–11, 10.1186/s12903-023-02879-2 10.1186/s12903-023-02879-2 PMC10064678 36997970

[7] Keul C, Liebermann A, Schmidlin PR, Roos M, Sener B, Stawarczyk B (2014). Influence of PEEK surface modification on surface properties and bond strength to veneering resin composites. J Adhes Dent Aug.

[8] Zhou L, Qian Y, Zhu Y, Liu H, Gan K, Guo J. The effect of different surface treatments on the bond strength of PEEK composite materials. (2014) Dent Mater. 1;30(8): e209–15, 10.1016/j.dental.2014.03.011 10.1016/j.dental.2014.03.011 24768752

[9] Schmidlin P, Stawarczyk B, Wieland M, Attin T, Hämmerli, Fischer J (2010). Effect of different surface pre-treatments and luting materials on shear bond strength to PEEK. Dent Mater.

[10] Stawarczyk B, Keul C, Beuer F, Roos M, Schmidlin PR (2013). Tensile bond strength of veneering resins to PEEK: impact of different adhesives. Dent Mater J.

[11] Stawarczyk B, Taufall S, Roos M, Schmidlin PR, Lümkemann N. Bonding of composite resins to PEEK: the influence of adhesive systems and air-abrasion parameters(2018) Clin Oral Investig. Mar 1;22(2):763–71., 10.1007/s00784-017-2151-x 10.1007/s00784-017-2151-x 28647864

[12] Stawarczyk B, Beuer F, Wimmer T, Jahn D, Sener B, Roos M (2013). Polyetheretherketone-a suitable material for fixed dental prostheses?. J Biomed Mater Res B Appl Biomater.

[13] Stawarczyk B, Jordan P, Schmidlin PR, Roos M, Eichberger M, Gernet W (2014). PEEK surface treatment effects on tensile bond strength to veneering resins. J Prosthet Dent.

[14] Taha D, Safwat F, Wahsh M. Effect of combining different surface treatments on the surface characteristics of polyetheretherketone-based core materials and shear bond strength to a veneering composite resin. (2022) J Prosthet Dent.;127(4): 599.e1-599.e7, 10.1016/j.prosdent.2022.01.004 10.1016/j.prosdent.2022.01.004 35135675

[15] Stawarczyk B, Bähr N, Beuer F, Wimmer T, Eichberger M, Gernet W (2014). Influence of plasma pretreatment on shear bond strength of self-adhesive resin cements to polyetheretherketone. Clin Oral Invest.

[16] Binhasan M, Alhamdan MM, Al-Aali KA, Vohra F, Abduljabbar T (2022). Shear bond characteristics and surface roughness of poly-ether-ether-ketone treated with contemporary surface treatment regimens bonded to composite resin. Photodiagnosis Photodyn Ther.

[17] Ates SM, Caglar I, Yesil Duymus Z. The effect of different surface pretreatments on the bond strength of veneering resin to polyetheretherketone(2018). J Adhes Sci Technol;32(20):2220–31, 10.1080/01694243.2018.1468534

[18] Gama LT, Duque TM, Özcan M, Philippi AG, Mezzomo LAM, Gonçalves TMSV (2020). Adhesion to high-performance polymers applied in dentistry: a systematic review. Dent Mater.

[19] Soares Machado P, Cadore Rodrigues AC, Chaves ET, Susin AH, Valandro LF, Pereira GKR (2022). Surface treatments and adhesives used to increase the Bond Strength between Polyetheretherketone and Resin-based Dental materials: a scoping review. J Adhes Dent.

[20] Kern M, Lehmann F (2012). Influence of surface conditioning on bonding to polyetheretherketon (PEEK). Dent Mater.

[21] Lümkemann N, Eichberger M, Stawarczyk B. Bond strength between a high-performance thermoplastic and a veneering resin. (2020) J Prosthet Dent Dec;124(6):790–7., 10.1016/j.prosdent.2019.10.017 10.1016/j.prosdent.2019.10.017 31980203

[22] Zarone F, Di Mauro MI, Ausiello P, Ruggiero G, Sorrentino R (2019). Current status on lithium disilicate and zirconia: a narrative review. BMC Oral Heal.

[23] Dahiya M, Tomer MS, Duhan VK. Bioactive glass/glass ceramics for dental applications. Appl Nanocomposite Mater Dent. 2019;11–25. 10.1016/B978-0-12-813742-0.00001-8

[24] Skorulska A, Piszko A, Rybak P, Szymonowicz Z, Dobrzyński M (2021). Review on Polymer, Ceramic and Composite materials for CAD/CAM indirect restorations in Dentistry-Application, mechanical characteristics and comparison. Mater (Basel).

[25] Faul F, Erdfelder E, Lang AG, Buchner A (2007). G*Power 3: a flexible statistical power analysis program for the social, behavioral, and biomedical sciences. Behav Res Methods.

[26] ISO 10477:2020. - Dentistry — Polymer-based crown and veneering materials. https://www.iso.org/standard/80007.html (accessed Jun. 14, 2023).

[27] Reda R, Zanza A, Galli M, De Biase A, Testarelli L, Di Nardo D (2022). Applications and clinical behavior of BioHPP in Prosthetic Dentistry: a short review. J Compos Sci.

[28] Vichi A, Zhao Z, Paolone G, Scotti N, Mutahar M, Goracci C, Louca C (2022). Factory Crystallized silicates for monolithic metal-free restorations: a Flexural Strength and Translucency Comparison Test. Materials.

[29] grò G, Rodi D, Sachs A, Hashimoto M. Modulus of elasticity of two ceramic materials and stress-inducing mechanical deformation following fabrication techniques and Adhesive Cementation procedures of a Dental Ceramic. Int J Biomater. 2019;4325845. 10.1155/2019/4325845 10.1155/2019/4325845 PMC6885839 31827519

[30] Sloan R, Hollis W, Selecman A, Jain V, Versluis A (2022). Bond strength of lithium disilicate to polyetheretherketone. J Prosthet Dent.

[31] Tosun B, Yanıkoğlu N (2022). Evaluation of the effects of different surface modification methods on the bond strength of high-performance polymers and resin matrix ceramics. Clin Oral Investig.

[32] Çulhaoğlu AK, Özkır SE, Şahin V, Yılmaz B, Kılıçarslan MA (2020). Effect of various treatment modalities on surface characteristics and Shear Bond strengths of Polyetheretherketone-based core materials. J Prosthodont.

[33] Chaijareenont P, Prakhamsai S, Silthampitag P, Takahashi H, Arksornnukit M (2018). Effects of different sulfuric acid etching concentrations on PEEK surface bonding to resin composite. Dent Mater J.

[34] Uhrenbacher J, Schmidlin PR, Keul C, Eichberger M, Roos M, Gernet W, Stawarczyk B (2014). The effect of surface modification on the retention strength of polyetheretherketone crowns adhesively bonded to dentin abutments. J Prosthet Dent.

[35] Gouveia DDNM, Razzoog ME, Sierraalta M, Alfaro MF (2022). Effect of surface treatment and manufacturing process on the shear bond strength of veneering composite resin to polyetherketoneketone (PEKK) and polyetheretherketone (PEEK). J Prosthet Dent.

[36] Gale MS, Darvell BW (1999). Thermal cycling procedures for laboratory testing of dental restorations. J Dent.

[37] Jahandideh Y, Falahchai M, Pourkhalili H. Effect of Surface Treatment with Er: YAG and CO2 lasers on Shear Bond Strength of Polyether Ether Ketone to Composite Resin veneers. J Lasers Med Sci. 2020 Spring;11(2):153–9. 10.34172/jlms.2020.26 10.34172/jlms.2020.26 PMC7118504 32273956

[38] Ourahmoune R, Salvia M, Mathia TG, Mesrati N (2014). Surface morphology and wettability of sandblasted PEEK and its composites. Scanning.

[39] Hallmann L, Mehl A, Sereno N, Hämmerle CH. F. *The improvement of adhesive properties of PEEK through different pre-treatments. Applied Surface Science 258 (*2012): 7213–7218. 10.1016/j.apsusc.2012.04.040

[40] Rosentritt M, Preis V, Behr M, Sereno N, Kolbeck C (2015). Shear bond strength between veneering composite and PEEK after different surface modifications. Clin Oral Investig.

[41] Lümkemann N, Strickstrock M, Eichberger M, Zylla IM, Stawarczyk B. Impact of air-abrasion pressure and adhesive systems on bonding parameters for polyetheretherketone dental restorations. Int J Adhes Adhes 2018 1; 80:30–8. 10.1016/j.ijadhadh.2017.10.002

[42] Malysa A, Wezgowiec J, Grzebieluch W, Danel DP, Wieckiewicz M (2022). Effect of Thermocycling on the Bond Strength of Self-Adhesive Resin cements used for luting CAD/CAM Ceramics to Human Dentin. Int J Mol Sci.

[43] Sproesser O, Schmidlin PR, Uhrenbacher J, Roos M, Gernet W, Stawarczyk B (2014). Effect of sulfuric acid etching of polyetheretherketone on the shear bond strength to resin cement. J Adhes Dent.

